# Limb Body Wall Complex with Sacrococcygeal Mass and Agenesis of External Genitalia

**DOI:** 10.1155/2013/218626

**Published:** 2013-07-22

**Authors:** Prabahita Baruah, Pradipta Ray Choudhury

**Affiliations:** ^1^Department of Anatomy, Fakhruddin Ali Ahmed Medical College, Barpeta-781301, Assam, India; ^2^M-28, Pragjyotish Housing Complex, Khanka Path, Ghoramara, Guwahati, Assam 781028, India

## Abstract

Limb body wall complex (LBWC) is a rare clinicopathological entity, characterized by the presence of an abdominal wall defect associated with variable spectrum of limb and visceral anomalies. A stillborn baby of LBWC with placentoabdominal phenotype is reported here. Kyphoscoliosis, sacrococcygeal mass and agenesis of external genitalia are the associated features.

## 1. Introduction

Limb-body wall complex (LBWC) is a rare, polymalformative fetal syndrome, appearing in 0.21–0.31/10000 deliveries [1, 2]. The diagnosis of this entity is based on two of the three following characteristics: (1) exencephaly/encephalocele and facial clefts; (2) thoraco- and/or abdominoschisis; and (3) limb defects [3].

Limb-body wall malformations result from a malfunction of the ectodermal placodes involving the early embryonic folding process [3]. The chance of recurrence in the next pregnancy is very low. However, the association of other malformations along with body wall defect makes the prognosis very poor [4]. The poor prognosis of LBWC calls for early antenatal diagnosis [3].

To the best of our knowledge, very few cases of LBWC in a stillborn fetus have been reported from India.

## 2. Case Presentation

A stillborn baby with birth weight 2700 gm was collected from Department of Obstetrics and Gynaecology, Fakhruddin Ali Ahmed Medical College and Hospital, Barpeta, Assam. External examination showed a large median defect in the anterior abdominal wall with loops of intestine coming out of the defect. There was no covering sac. 

**Figure 1:**
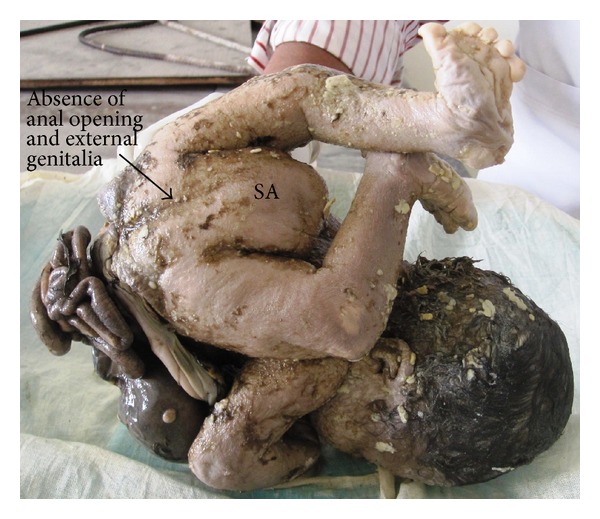
Showing sacrococcygeal mass (SA) and absence of anal opening and external genitalia.

**Figure 2:**
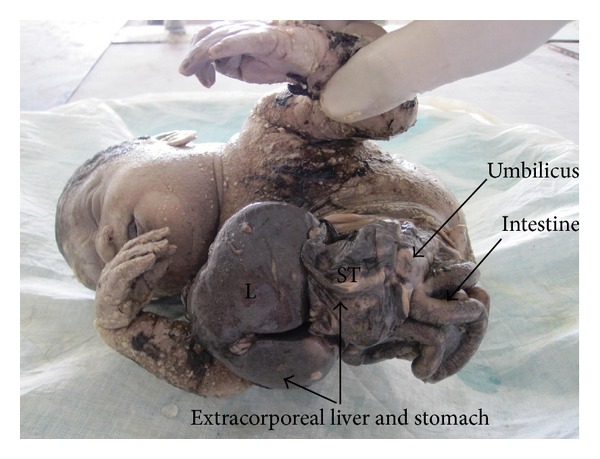
Showing abdominoschisis with evisceration of stomach, intestine, and extracorporeal liver.

**Figure 3:**
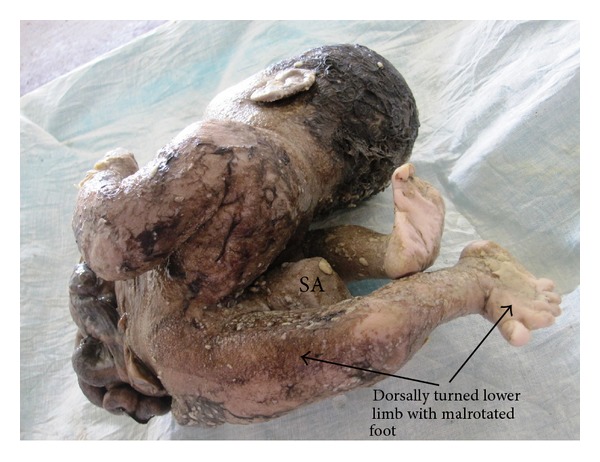
Showing dorsally rotated lower limbs and malrotated foot.

The abdominoschisis had complete evisceration of the stomach, bowel, and extracorporeal liver (stomach marked as “ST,” liver as “L” on Figure 2). A sacrococcygeal mass (sacrococcygeal mass marked as “SA” on Figure 1) was located on the posteroinferior part of the trunk and gluteal region and was completely external. Both lower limbs were dorsally turned with malrotated foot (shown in Figure 3). There was no discernable external genitalia. Anal atresia was present (shown in Figure 1).

Placenta was normal, but umbilical cord was short with presence of two umbilical arteries and one umbilical vein, and the cord was situated in the middle of the extruded abdominal contents. Kyphoscoliosis was present.

## 3. Discussion

The limb-body wall complex is also known as the body-stalk syndrome. It is a rare entity characterized by severe malformations. Most fetuses are aborted, either spontaneously or by medical means. Most of the remaining are stillborn [5].

The diagnostic criteria for LBWC are still being discussed, but the most commonly quoted are those originally set forth by Van Allen et al. in 1987, that is, the presence of two of the following three malformations: (a) exencephaly/encephalocele and facial clefts; (b) thoraco- and/or abdominoschisis; and (c) limb defects [6]. A definite association with internal anomalies and severe kyphoscoliosis makes a more distinct concept of the pathogenesis reasonable [3]. In our case, abdominoschisis was present with limb defects and kyphoscoliosis.

Russo et al. identified two distinct phenotypes of LBWC, one with craniofacial defects, facial clefts, amniotic adhesions, and amniotic band sequences called placentocranial adhesion phenotype and the other without craniofacial defects but with imperforate anus, urogenital abnormalities, lumbosacral meningomyeclocle, and kyphoscoliosis called the placentoabdominal adhesion phenotype [7]. Our case resembles the placentoabdominal phenotype.

Congenital malformations of the ventral abdominal wall occur in many forms, ranging from exomphalos to gastroschisis to more complex malformations, such as pentalogy of Cantrell and LBWC [4].

Gastroschisis is a small full-thickness defect of the anterior abdominal wall, usually just to the right of umbilicus [4]. In our study, the cord was situated in the middle of the extruded abdominal contents.

Exomphalos involves herniation of abdominal viscera, through an enlarged umbilical ring, covered by amnion [8]. But in the present study, herniated abdominal content is not covered by amnion.

Pentalogy of Cantrell includes ectopia cordis, defects in anterior region of the diaphragm, absence of the pericardium, defects in the sternum and abdominal wall, and defects including exomphalos and gastroschisis [9]. 

Three major theories have been suggested to explain this complex: early amnion rupture (operating through uterine pressure and/or disruption by amniotic bands), vascular compromise (primarily hypoperfusion), and an early intrinsic defect of the developing embryo [10].

There is no correlation of LBWC with the fetal gender, parents' age, or karyotype anomalies [6].

## 4. Conclusion

The prognosis of LBWC is very poor compared to isolated exomphalos or gastroschisis. Pregnancy should be terminated on establishing correct diagnosis, which requires careful ultrasound of the fetus whenever ventral abdominal wall defect is suspected.
